# Characteristics of Sepsis-2 septic shock patients failing to satisfy the Sepsis-3 septic shock definition: an analysis of real-time collected data

**DOI:** 10.1186/s13613-021-00942-1

**Published:** 2021-10-30

**Authors:** Joris Vermassen, Johan Decruyenaere, Liesbet De Bus, Pieter Depuydt, Kirsten Colpaert

**Affiliations:** 1 Department of Intensive Care Medicine, University Hospital Gent, 2K12C. C. Heymanslaan 10, 9000 Gent, Belgium; 2 Faculty of Medicine and Health Sciences, University Gent, Gent, Belgium

**Keywords:** Septic shock, Intensive care unit, Epidemiology, Lactate, Chronic liver disease

## Abstract

**Background:**

Baseline characteristics and disease severity of patients with septic shock according to the new Sepsis-3 definition may differ from patients that only comply with the Sepsis-2 definition. We conducted a retrospective cohort study on the ICU of a Belgian tertiary care facility to seek out differences between these two patient groups and to identify variables associated with no longer satisfying the latest definition of septic shock.

**Results:**

Of 1198 patients with septic shock according to the Sepsis-2 consensus definition, 233 (19.4%) did not have septic shock according to the Sepsis-3 shock definition. These patients more often had medical admission reasons and a respiratory infection as cause for the septic shock. They less often had surgery on admission and were less likely to have chronic liver disease (5.6% vs 16.2%, absolute difference 10.6% (95% CI 6.4–14.1%). Patients with septic shock only according to the old definition had significant lower APACHE II and SOFA scores and lower hospital mortality (31.6% vs 55.3%, *p* < 0.001). In a multivariate analysis, following variables were associated with Sepsis-2 shock patients no longer being defined as such by the Sepsis-3 definition: respiratory infection (OR 1.485 (95% CI 1.56–2.089), *p* = 0.023), a medical admission reason (OR 1.977 (95% CI 1.396–2.800) and chronic liver disease (OR 0.345 (95% CI 0.181–0.660), *p* < 0.001).

**Conclusions:**

One in five patients with septic shock according to the Sepsis-2 consensus definition is no longer considered as such when the Sepsis-3 shock criteria are applied. A medical admission reason, a respiratory infection and absence of chronic liver disease are independently associated with no longer being identified as having septic shock by the Sepsis-3 criteria.

**Supplementary Information:**

The online version contains supplementary material available at 10.1186/s13613-021-00942-1.

## Background

In 2016 a new consensus definition of sepsis and septic shock was published. Patients are required to have sepsis (a proven or suspected infection in combination with a rise in sequential organ failure assessment score (SOFA) of at least 2 points compared to baseline), persistent hypotension requiring vasopressor therapy to maintain a mean arterial pressure of at least 65 mm of mercury (Hg) and a lactate of at least 2 mmol/l despite adequate volume resuscitation to be considered to have septic shock according to this new consensus definition [1]. In contrast, for patients to comply with the previous Sepsis-2 consensus definition, they needed to have sepsis (defined as a proven or suspected infection in combination with at least 2 systemic inflammatory response syndrome (SIRS) criteria) and persistent hypotension (defined as a mean arterial pressure below 60 mm Hg, a systolic blood pressure below 90 mm Hg or a decrease in systolic blood pressure of at least 40 mm Hg) despite adequate fluid resuscitation [2]. Ever since, the effect of the change of the definition on patient selection has been scrutinized. Some authors report lower sensitivity but higher specificity in detecting patients with septic shock when the new criteria are applied, as well as higher mortality in patients they identify [3, 4]. The use of SOFA or quick SOFA (qSOFA) also results in a more accurate prediction of risk of mortality compared to the old definitions [5, 6]. Consequently, a certain number of patients being classified as having sepsis or septic shock according to this old definition will no longer be held as such by the new one [7]. This could result in a subgroup of septic patients no longer receiving the official label “septic shock” and consequently being excluded from clinical trials or epidemiological studies with all consequences thereof. Our primary goal was to investigate baseline characteristics of septic shock patients who meet the old Sepsis-2 consensus definition but are no longer considered as such by the new Sepsis-3 septic shock definition—with lactate values below 2 mmol/l—and to describe differences in severity of illness. Additionally, we wanted to identify the factors that are associated with this exclusion.

## Methods

This study was performed at the Ghent University Hospital, a tertiary care facility with a 66-bed intensive care unit (ICU) containing a surgical, cardiothoracic, medical, pediatric and burn unit. During the study period, our ICU was 24/7 covered by at least one consultant intensive care physician and four intensive care residents. All infections in our ICU were prospectively recorded in our own ICU’s COSARA database. In COSARA (Computer-based Surveillance and Alerting of infections, Antimicrobial Resistance and Antibiotic consumption in the ICU) every infection was graded in severity according to the Sepsis-2 consensus definition [2, 8, 9]. Patients with septic shock were treated according to the surviving sepsis guidelines with norepinephrine as first choice vasopressor [10]. Data from all consecutively admitted patients between January 1st, 2013, and December 31st, 2018, with a diagnosis of septic shock according to COSARA and for whom a prescription for the administration of norepinephrine could be retrieved from the health record, were gathered. We only included patients admitted to the surgical or medical ICU. Patients without reported lactate value were excluded. If patients experienced a recurrent septic shock episode during the same hospitalization, only the first ICU admission was included. The remaining patients were considered to have septic shock according to the Sepsis-2 definition.

Starting from all patients in our database complying with the definition of septic shock according to the Sepsis-2 definition, we created two groups. The first group of patients was called “S3+ ”. These patients had, on top of vasopressor therapy, also a rise in lactate above 2 mmol/l at the same timepoint, which caused them to comply with the Sepsis-3 shock definition. The remaining patients only had vasopressor therapy, but had no reported lactate value above 2 mmol/l, so they did not satisfy the Sepsis-3 shock definition. These patients were called “S2+ /S3− “ [1].

Demographic data, comorbidities, infection characteristics, laboratory results, medication prescriptions, organ support variables and outcome parameters were collected from the electronic patient record (GE Healthcare, Centricity Critical Care). Charlson comorbidity index (CCI), SOFA score and Acute Physiology and Chronic Health Evaluation (APACHE II) score were calculated for every patient [11–13]. A medical admission reason was defined as a condition with need for admission in the ICU in patients with otherwise no present indication for surgical intervention.

Numeric variables are expressed as median and interquartile range, nominal variables are expressed as *n* (%). Differences between the S3+ and S2+ /S3− patients were assessed using Chi-square and Mann–Whitney U test where appropriate and statistical significance was attained at *p* < 0.05. Absolute differences between the two defined populations were calculated using the independent samples proportions test. A multivariate logistic regression model was constructed to identify variables independently associated with being excluded from the Sepsis-3 definition of septic shock. Therefore, all variables that were univariately associated with being excluded from the Sepsis-3 septic shock definition were used. Organ dysfunction variables were not used because we aimed to make a baseline risk analysis based only on variables that are possibly known at the moment of the onset of septic shock, in essence when the decision to start supportive therapy should be made.

All analyses were performed with IBM SPSS statistics v 26. Approval for this study was obtained from the local hospital’s ethical committee (EC/2016/0254) and the need for informed consent was waived.

## Results

**Fig. 1:**
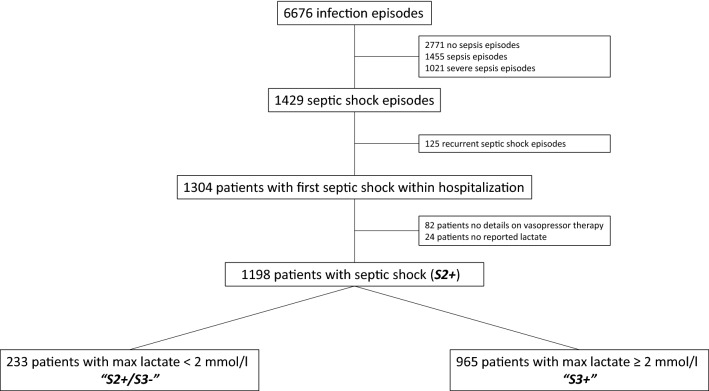
Patient selection

During the 6-year timeframe, 6676 infection episodes requiring antibiotic treatment were registered in COSARA. After exclusion of recurrent episodes of septic shock and of patients missing lactate levels or details on vasopressor therapy, 1198 patients were identified with septic shock according to the Sepsis-2 definition. A total of 233 patients (19.4%) did not have septic shock according to the Sepsis-3 definition (S2+ /S3−), 965 patients (80.6%) had septic shock both according to the Sepsis-2 and Sepsis-3 definition (S3+) (Fig. 1).

Baseline characteristics of the patients can be found in Table 1. S2+ /S3− patients more frequently had a medical admission reason and less surgery on admission. As to infection characteristics, S2+ /S3− patients had more respiratory infections causing septic shock, less bacteremia or positive cultures from other clinically significant sites. There was a notable difference in chronic liver disease (CLD) between the S2+ /3− and S3+ patients (13 (5.6%) vs 153 (16.2%), absolute difference 10.6% (95% CI 6.4–14.1%). Furthermore, patients from the S2+ /S3− group were more frequently active smokers and were associated more often with a history of hematological conditions or chronic pulmonary disease and had fewer malignancies (Table 2). Overall comorbidity burden was high with a median CCI of 4 both in the S3+ and S2+ /S3− group.

**Table 1:** Baseline characteristics of the study population

	S3+	S2+ /S3 −	Absolute difference	95% CI		*p*
				Lower bound	Upper bound	
Male	601 (62.3)	160 (68.7)	− 6.4	− 12.9	0.4	0.069
Age	64 (54–73)	64 (54–71)				0.818
Weight	75 (65–85)	76 (65–87)				0.095
Living at home	719 (83.7)	172 (83.1)	0.6	− 4.8	6.5	0.832
Admission						0.384
Other hospital	179 (19.0)	36 (15.7)				
Emergency department	348 (37.0)	94 (41.0)				
Ward	413 (43.9)	99 (43.2)				
Readmission < 48 h	22 (2.3)	9 (3.9)	− 1.6	− 4.6	0.9	0.172
Medical admission reason	466 (48.3)	155 (66.5)	− 18.2	− 24.9	− 11.3	< 0.001
Surgery	243 (25.3)	36 (15.5)	9.8	4.1	14.9	0.002
Respiratory septic shock	287 (29.7)	96 (41.2)	− 11.5	− 18.4	− 4.6	0.001
Abdominal septic shock	312 (32.3)	45 (19.3)	13.0	6.9	18.7	< 0.001
Bacteremia	262 (27.2)	43 (18.5)	8.7	2.8	14.2	0.006
Culture negative septic shock	362 (37.5)	110 (47.2)	− 9.7	− 16.8	− 2.6	0.007

All values are expressed as median (interquartile range) or n (%). Absolute differences are reported for percentages

**Table 2:** Comorbidities of the study population

				95% CI		*p*
	S3+	S2+ /S3–	Absolute difference	Lower bound	Upper bound	
Nicotine	194 (25.5)	67 (33.8)	− 8.3	− 15.7	− 1.1	0.019
Alcohol abuse	152 (18.5)	23 (11.3)	7.2	1.8	12.0	0.014
NYHA III/IV	242 (26.6)	70 (31.3)	− 4.7	− 11.5	1.9	0.159
Organ transplantation	19 (5.1)	8 (10.5)	− 5.4	− 13.6	1.3	0.074
Stem cell transplantation	7 (1.9)	0 (0)	1.9	− 2.0	3.8	0.226
Malignancy	174 (28.9)	50 (21.6)	7.3	1.0	13.1	0.026
Metastatic malignancy	100 (10.6)	18 (7.8)	2.8	− 1.5	6.5	0.209
Hematologic condition	111 (11.7)	41 (17.7)	− 6.1	− 11.6	− 0.9	0.013
Chronic Liver disease	153 (16.2)	13 (5.6)	10.6	6.4	14.1	< 0.001
Pulmonary disease	162 (17.2)	54 (23.3)	− 6.1	− 12.2	− 0.3	0.032
Chronic kidney disease	206 (21.7)	63 (27.3)	− 5.6	− 12.0	0.6	0.071
Patient on dialysis	24 (2.5)	11 (4.8)	− 2.2	− 5.5	0.5	0.073
Diabetes mellitus	202 (21.2)	48 (20.7)	0.5	− 5.5	6.2	0.854
Hypertension	305 (32.2)	78 (33.8)	− 1.6	− 8.5	5.1	0.643
Vascular disease	160 (16.9)	45 (19.5)	− 2.6	− 8.4	2.8	0.349
Ischemic stroke	50 (5.3)	11 (4.8)	0.5	− 3.0	3.4	0.753
Coronary disease	180 (19.1)	45 (19.4)	− 0.3	− 6.2	5.2	0.909

All values are expressed as median (IQR) or *n* (%). NYHA: New York Heart Association Scale

**Fig. 2:**
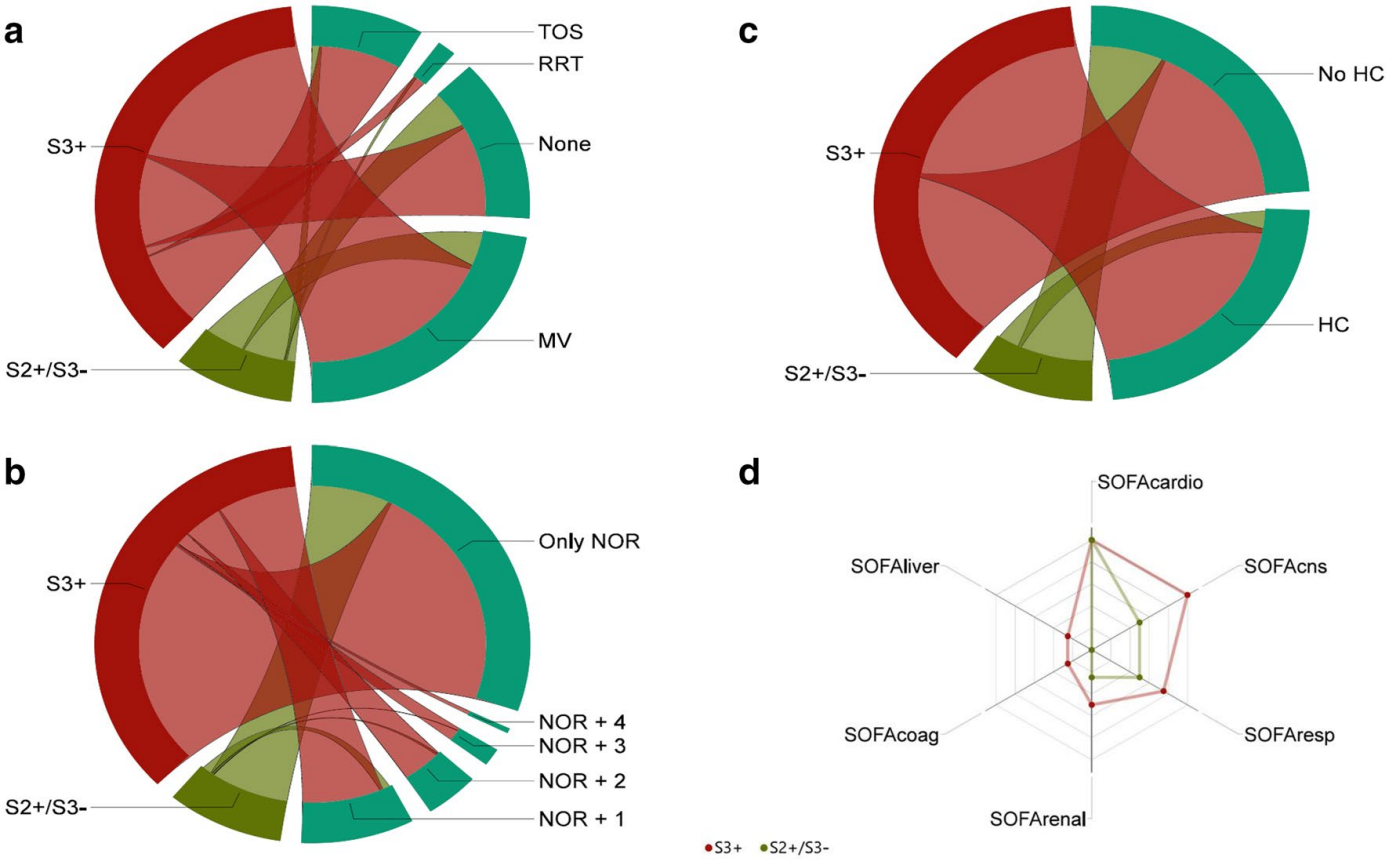
Differences in organ support and organ dysfunction variables between the S3+ and S2+ /S3− patients. **a** Chord diagram representing the need for organ support—on top of vasopressor therapy—in S3+ and S2+ /S3− patients. RRT: only renal replacement therapy, MV: only mechanical ventilation, TOS: triple organ support—combination of vasopressor therapy, MV and RRT. **b** Chord diagram representing administration of hydrocortisone (HC) in septic shock. **c** Chord diagram representing the number of adjuvant vasopressor or inotropic agents on top of norepinephrine (NOR). Examined medications are epinephrine, vasopressin, dobutamine and milrinone. **d** Radar plot of the SOFA subscores: cardiac (SOFAcardio), neurological (SOFAcns), respiratory (SOFAresp), renal (SOFArenal), coagulation (SOFAcoag) and hepatic (SOFAliver)

Details on organ support and severity of illness can be found in Fig. 2. Fewer patients in the S2+ /S3− group received mechanical ventilation (MV) (124 (53.2%) vs 689 (71.4%), absolute difference 18.2% (95% CI 11.2–25.2%), *p* < 0.001), received renal replacement therapy (RRT) (27 (11.6%) vs 227 (23.5%), absolute difference 11.9% (95% CI 6.7–16.6%), *p* < 0.001) or received triple organ support with a combination of vasopressor therapy, MV and RRT (23 (9.9%) vs 198 (20.5%), absolute difference 10.6% (95% CI 5.7–15.0%), *p* < 0.001) compared to S3+ patients. Additionally, they received less adjuvant vasopressor or inotropic support on top of norepinephrine, compared to S3+ patients. Maximal SOFA score in the S2+ /3+ group was 10 (8–12.5) compared to 13 (10–16) in S3+ patients (*p* < 0.001) and APACHE II score was 24 (18–30) and 29 (21–35), respectively. A detailed description of organ dysfunction variables can be found in Additional file 1: Tables S4 and S5 as well as in Additional file 1: Fig. S2.

In a multivariate analysis, following criteria were identified with being excluded from the Sepsis-3 septic shock definition (S2+ /S3− patients): respiratory infection as cause for septic shock (OR 1.485 (1.056–2.089) *p* = 0.023), medical admission reason (OR 1.977 (1.396–2.800) *p* < 0.001). A comorbidity of chronic liver disease was inversely associated (OR 0.345 (0.181–0.660) *p* < 0.001) (Table 3).

**Table 3:** Univariate and multivariate analysis of variables associated with exclusion from the Sepsis-3 definition of septic shock

	OR	95% CI	*p*
Univariate analysis			
Respiratory septic shock	1.655	1.232—2.224	0.001
Abdominal septic shock	0.501	0.352—0.712	< 0.001
Surgery on admission	0.543	0.370—0.798	0.002
Medical admission reason	2.128	1.576—2.872	< 0.001
Nicotine	1.492	1.066—2.089	0.02
Alcohol abuse	0.561	0.351—0896	0.016
Malignancy	0.679	0.481—0.956	0.027
Hematological condition	1.633	1.104—2.415	0.014
Chronic liver disease	0.307	0.171—0.552	< 0.001
Chronic pulmonary disease	1.461	1.031—2.069	0.033
Bacteremia	0.607	0.424—0.870	0.007
Culture negative septic shock	1.49	1.117—1.988	0.007
Multivariate analysis			
Respiratory septic shock	1.485	1.056—2.089	0.023
Medical admission reason	1.977	1.396—2.800	< 0.001
Chronic liver disease	0.345	0.181—0.660	0.001

ICU and hospital mortality were significantly lower in the S2+ /S3− group (43 (19.4%) and 65 (31.6%) vs 416 (43.9%) and 501 (55.3%), *p* < 0.001). In the baseline cohort of patients complying with the Sepsis-2 consensus definition, hospital mortality in patients with CLD was 71.4%, compared to 47.2% in patients without (*p* < 0.001). In patients with CLD, mortality is higher if they comply with the Sepsis-3 definition than if they only comply with the Sepsis-2 definition (75.0% vs 30.8%, *p* < 0.001) (Additional file 1: Tables S8 and S9).

## Discussion

We compared characteristics of patients who met the Sepsis-2 shock criteria but not the Sepsis-3 shock definition (S2+ /S3−) with those that complied with both septic shock definitions (S3+). In our cohort, 19.4% of septic shock patients meeting the Sepsis-2 definition did not comply with the Sepsis-3 shock definition. This number is lower than figures reported by other authors [14–18]. A possible explanation is that our patient selection was based on the labeling of septic shock in the COSARA registry, which also necessitates the administration of norepinephrine, whereas other publications have larger proportions of septic shock patients who did not receive vasopressor therapy [15].

In our trial, we are essentially comparing 2 groups of patients that receive vasopressor therapy but that differ in lactate levels, in that sense that S2+ /S3− patients never had a lactate level of 2 mmol/l or above during vasopressor therapy. This lactate criterion is the most important difference between the Sepsis-2 and Sepsis-3 consensus shock criteria [1, 2]. Lactate levels have been used as a marker of tissue hypoperfusion, with all limitations thereof since these are influenced by several mechanisms of lactate production (severity of shock, ischemia, administration of beta adrenergic medication, malignancies) and by lactate metabolism which is impaired in CLD [19]. Moreover, lactate levels are highly variable and must by consequence be interpreted as a snapshot in the course of the disease. Patients with higher lactate levels—and presumably with a higher degree of tissue hypoperfusion—in our cohort have higher severity scores and higher need for organ supportive therapy. This adds further support for the new septic shock definitions identifying a sicker cohort of patients with higher risk of mortality [1, 14–16, 20].

Three baseline characteristics were independently associated with not being included in the latest consensus definition of septic shock. First of all, excluded patients had more medical admission reasons compared to surgical admission reasons. We hypothesize that several medical admission reasons (like several inflammatory syndromes in hematological or immunosuppressed patients or after circulatory arrest that are nonetheless treated with antibiotics because there is doubt concerning an underlying infection) could be associated less with tissue hypoperfusion and lactate production compared with surgical admission reasons like peritonitis. This hypothesis is supported by the significant difference in abdominal infections and positive cultures between the S2+ /S3− and S3+ patients. Secondly, more patients in the S2+ /S3− group had a respiratory infection as cause for the septic shock. It is conceivable that these patients need more sedation to accommodate mechanical ventilation, and this may cause a higher need for vasopressor therapy which is not necessarily associated with shock and hypoperfusion. At last, the proportion of patients with CLD was three times higher in the S3+ group compared to the S2+ /S3− group. This can be explained by reduced lactate clearance in patients with CLD, which may be more profound in the presence of sepsis—even in cases of mild liver disease. Patients with CLD also have higher ICU and hospital mortality compared to patients with normal hepatic function [19, 21–24] (Additional file 1: Tables S6–S8).

The lower mortality in the S2+ /S3− group compared to the S3+ group (31.6% vs 55.3%) is consistent with what other authors have reported [1, 3, 14–16, 20] while other authors report a similar mortality in both groups [4]. Mortality rates in our study were higher than what is found in the literature. This may be due to the high severity of the acute illness in our study population and to the high comorbidity burden (median CCI 4 in both groups) [14, 15, 25].

When the new criteria for septic shock are applied for research purposes, the abovementioned patients are at risk of being excluded. By consequence, a different group of patients may be defined compared to trials using the Sepsis-2 criteria. This may introduce a gap in continuity of research in the field of septic shock. This is especially the case if the consensus definitions are systematically used for trial inclusion. However, when reviewing randomized controlled trials in septic shock, there is a vast heterogenicity in inclusion criteria which may have, not surprisingly, an effect on mortality figures [26]. This further complicates comparison between different interventional trials since the patients that are investigated differ to a greater or lesser extent. This effect is also seen in retrospective trials. When comparing several groups of patients with different criteria like need for vasopressor therapy, need for a certain lactate level or need for certain blood pressure targets—that can all be considered to be septic shock in one way or another, depending on the definition used—one notices a difference in number of patients pertaining to these groups but also a difference in mortality. This points out the important between-group variance [17, 18].

This was already noted by the authors of the third consensus conference definition who tried to create order in the existing chaos by introducing sound and new criteria to define septic shock, by selecting patients based on two objective criteria: clinically significant hypotension (and so need for vasopressor therapy) and tissue hypoperfusion (reflected by a rise in lactate above 2 mmol/l) [1, 20]. But no matter which criteria are used, in order to be able to comprehend, compare and discuss septic shock literature, it is important everyone speaks the same language, which means using a single set of diagnostic criteria worldwide.

Our study has some limitations: it has a monocentric retrospective design so the conclusions that apply to our tertiary patient population may not completely apply to other hospitals. The data used were primarily gathered for patient care, not for scientific research. Quality of the collected data depends, in no small amount, on correct prescriptions of medication or correct registration of parameters. Almost all data on baseline characteristics or comorbidity were collected at the time of admission to the ICU. The rate of missing data for the majority of variables was less than 5%. For a few valuables like smoking and alcohol abuse, however, missing data rate could go as high as 14 to 20%. Since the value of the Glasgow Coma Scale before patients are sedated is not always recorded, SOFA and APACHE II scores may be overestimated, but still reflect the high severity of illness in our cohort. The data are gathered entirely from the ICU Patient Data Management System. Since this is not used on normal wards or in the emergency department, data on vasopressor use or fluid therapy prior to admission on the ICU are not available.

## Conclusion

One in five patients with septic shock according to the previous septic shock definition was no longer considered as such when the new Sepsis-3 septic shock criteria were applied. A medical admission reason, a respiratory focus of the septic shock and absence of chronic liver disease are associated with decreased probability of being defined as having septic shock according to the Sepsis-3 definition in our cohort.

## Supplementary Information

**Additional file 1: Table S1.** Distribution of the source of infection in S3+ and S2+/S3− patients. **Figure S1.** Distribution of the source of infection in S3+ and S2+/S3− patients. **Table S2.** Baseline characteristics of the study population: extended. **Table S3.** Comorbidity characteristics: extended. **Table S4.** Organ support variables. **Table S5.** Maximal and minimal values of several organ dysfunction variables. **Figure S2.** Comparison of several organ dysfunction variable between S3+ and S2+/S3−. Figure S3: Evolution of mortality of S3+ patients. **Table S6.** Comparison between septic shock patients with or without medical admission reason. **Table S7.** Comparison between septic shock patients with or without respiratory infection. **Table S8.** Comparison between septic shock patients with or without chronic liver disease. **Table S9.** Comparison in patients fulfilling Sepsis-3 criteria and patients not fulfilling Sepsis-3 criteria in the subgroup of patients with chronic liver disease. **Figure S4.** Proportion of S3+ patients and mortality in function of lactate levels. **Figure S5.** Proportion of patients with chronic liver disease in the S2+/S3− and S3+ group in function of lactate levels.

## Data Availability

The datasets used during the current study are available from the corresponding author on reasonable request.

## Abbreviations

SOFA: Sequential Organ Failure Assessment
Hg: Mercury
SIRS: Systemic inflammatory response syndrome
qSOFA: Quick Sequential Organ Failure Assessment
ICU: Intensive care unit
COSARA: Computer-based surveillance and alerting of infections, antimicrobial resistance and antibiotic consumption in the ICU
CCI: Charlson comorbidity index
APACHE II: Acute Physiology and Chronic Health Evaluation II
CLD: Chronic liver disease
MV: Mechanical ventilation
RRT: Renal replacement therapy
