# Supplementary material for: Characteristics of Sepsis-2 septic shock patients failing to satisfy the Sepsis-3 septic shock definition: an analysis of real-time collected data
Source: Ann Intensive Care. 2021 Oct 30;11:154. doi: 10.1186/s13613-021-00942-1 (PMC8557229; doi:10.1186/s13613-021-00942-1)
Supplement: Supplementary file 1 — Additional file 1: Table S1. Distribution of the source of infection in S3+ and S2+/S3− patients. Figure S1. Distribution of the source of infection in S3+ and S2+/S3− patients. Table S2. Baseline characteristics of the study population: extended. Table S3. Comorbidity characteristics: extended. Table S4. Organ support variables. Table S5. Maximal and minimal values of several organ dysfunction variables. Figure S2. Comparison of several organ dysfunction variable between S3+ and S2+/S3−. Figure S3: Evolution of mortality of S3+ patients. Table S6. Comparison between septic shock patients with or without medical admission reason. Table S7. Comparison between septic shock patients with or without respiratory infection. Table S8. Comparison between septic shock patients with or without chronic liver disease. Table S9. Comparison in patients fulfilling Sepsis-3 criteria and patients not fulfilling Sepsis-3 criteria in the subgroup of patients with chronic liver disease. Figure S4. Proportion of S3+ patients and mortality in function of lactate levels. Figure S5. Proportion of patients with chronic liver disease in the S2+/S3− and S3+ group in function of lactate levels. [file 13613_2021_942_MOESM1_ESM.docx]

**Additional material**

Table S1: Distribution of the source of infection in S3+ and S2+/S3- patients.

Figure S1: Distribution of the source of infection in S3+ and S2+/S3- patients.

Table S2: Baseline characteristics of the study population: extended.

Table S3: Comorbidity characteristics: extended.

Table S4: Organ support variables.

Table S5: Maximal and minimal values of several organ dysfunction varables.

Figure S2: Comparison of several organ dysfunction variable between S3+ and S2+/S3-.

Figure S3: Evolution of mortality of S3+ patients.

Table S6: Comparison between septic shock patients with or without medical admission reason.

Table S7: Comparison between septic shock patients with or without respiratory infection.

Table S8: Comparison between septic shock patients with or without chronic liver disease.

Table S9: Comparison in patients fullfilling Sepsis-3 citeria and patients not fullfilling Sepsis-3 criteria in the subgroup of patients with chronic liver disease.

Figure S4: Proportion of S3+ patients and mortality in function of lactate levels.

Figure S5: Proportion of patients with chronic liver disease in the S2+/S3- and S3+ group in function of lactate levels

**Table S1: Distribution of the source of infection in S2+/S3+ and S2+/S3- patients.**

| **Focus of infection** | **S3+** | **S2+/S3-** | **p = 0.004** |
| --- | --- | --- | --- |
| Respiratory | 287 (29.7) | 96 (41.2) |  |
| Abdominal | 312 (32.3) | 45 (19.3) |  |
| Other/unknown | 160 (16.6) | 38 (16.3) |  |
| Urogentital | 77 (8.0) | 15 (6.4) |  |
| Neuropenic sepsis | 47 (4.9) | 14 (6.0) |  |
| Bone/soft tissue | 41 (4.2) | 14 (6.0) |  |
| Bloodstream | 36 (3.7) | 9 (3.9) |  |
| Neurologic | 5 (0.5) | 2 (0.9) |  |

Values are presented as n (%).

**Figure S1: Distribution of the source of infection in S3+ and S2+/S3- patients.**

Distribution of infections in S3+ patients

Distribution of infection in S2+/S3- patients

**Table S2: Baseline characteristics of the study population: extended.**

|  | | | | **95%CI** | |  |
| --- | --- | --- | --- | --- | --- | --- |
|  | **S3+** | **S2+/S3-** | **Absolute difference** | **lower bound** | **upper bound** | **p** |
| **Male** | 601 (62.3) | 160 (68.7) | -6.4 | -12.9 | 0.4 | 0.069 |
| **Age (y)** | 64 (54 - 73) | 64 (54 - 71) |  | | | 0.818 |
| **18 - 64 y/o** | 501 (51.9) | 119 (51.1) | 0.8 | -6.3 | 8 | 0.817 |
| **65 - 79 y/o** | 382 (39.6) | 99 (42.5) | -2.9 | -10.0 | 4.1 | 0.417 |
| **> 79 y/o** | 82 (8.5) | 15 (6.4) | 2.1 | -1.9 | 5.4 | 0.301 |
| **Weight (kg)** | 75 (65 - 85) | 76 (65 - 87) |  | | | 0.095 |
| **Length (cm)** | 170 (165 - 178) | 171 (166 - 180) |  |  |  | 0.111 |
| **BMI** | 24.8 (22.1 - 28.4) | 25.1 (22.5 - 28.4) |  |  |  | 0.280 |

Values are presented as n (%) or median (IQR)

|  | | | | | **95%CI** | |  |
| --- | --- | --- | --- | --- | --- | --- | --- |
|  |  | **S3+** | **S2+/S3-** | **Absolute difference** | **lower bound** | **upper bound** | **p** |
| **Living at home** |  | 719 (83.7) | 172 (83.1) | 0.6 | -4.8 | 6.5 | 0.832 |
| **Nursing home resident** |  | 111 (12.9) | 25 (12.1) | 0.8 | -4.4 | 5.6 | 0.744 |
| **Admission pathway** |  |  |  |  | | | 0.384 |
|  | Other hospital | 179 (19.0) | 36 (15.7) |  |  |  |  |
|  | Emergency department | 348 (37.0) | 94 (41.0) |  |  |  |  |
|  | Ward | 413 (43.9) | 99 (43.2) |  |  |  |  |
| **Surgery prior to admission** |  |  |  |  |  |  | 0.007 |
|  | No surgery | 719 (74.7) | 196 (84.5) |  |  |  |  |
|  | Emergency surgery | 154 (16.0) | 22 (9.5) |  |  |  |  |
|  | Elective surgery | 89 (9.3) | 14 (6.0) |  |  |  |  |
| **Cardiac arrest** |  | 77 (8.0) | 10 (4.3) | 3.7 | -0.1 | 6.4 | 0.052 |
| **Medical ICU admission** |  | 466 (48.3) | 155 (66.5) | -18.2 | -24.9 | -11.3 | <0.001 |
| **Readmission < 48h** |  | 22 (2.3) | 9 (3.9) | -1.6 | -4.6 | 0.9 | 0.172 |
| **Delay admission - start antibiotics (h)** |  | 1.4 (0.7 - 4.8) | 1.5 (0.7 - 5.4) |  | | | 0.566 |
| **Delay admission - start vasopressor therapy (h)** |  | 1.3 (0.4 - 5.3) | 2.3 (0.7 - 9.5) |  |  |  | <0.001 |
| **Respiratory septic shock** |  | 287 (29.7) | 96 (41.2) | -11.5 | -18.4 | -4.6 | 0.001 |
| **Abdominal septic shock** |  | 312 (32.3) | 45 (19.3) | 13.0 | 6.9 | 18.7 | <0.001 |
| **Bacteremia** |  | 262 (27.2) | 43 (18.5) | 8.7 | 2.8 | 14.2 | 0.006 |
| **Culture negative septic shock** |  | 362 (37.5) | 110 (47.2) | -9.7 | -16.8 | -2.6 | 0.007 |
| **APACHE II** |  | 29 (21-35) | 24 (18-30) |  | | | <0.001 |
| **SOFA admission** |  | 10 (7-12) | 8 (6-11) |  |  |  | <0.001 |
| **SOFA max** |  | 13 (10-16) | 10 (8-12.5) |  |  |  | <0.001 |

Values are presented as n (%) or median (IQR)

**Table S3: Comorbidity characteristics: extended.**

|  |  |  |  | **95%CI** | |  |
| --- | --- | --- | --- | --- | --- | --- |
|  | **S3+** | **S2+/S3-** | **Absolute difference** | **lower bound** | **upper bound** | **p** |
| **Smoking** | 194 (25.5) | 67 (33.8) | -8.3 | -15.7 | -1.1 | 0.019 |
| **Alcohol abuse** | 152 (18.5) | 23 (11.3) | 7.2 | 1.8 | 12.0 | 0.014 |
| **Benzodiazepine use** | 80 (22.7) | 18 (25.0) | -2.3 | -13.7 | 8.1 | 0.677 |
| **NYHA III/IV** | 242 (26.6 | 70 (31.3) | -4.7 | -11.5 | 1.9 | 0.159 |
| **Chair bound/bedridden** | 131 (14.1) | 28 (12.3) | 1.8 | -3.3 | 6.4 | 0.485 |
| **Active immunesuppressive condition** | 188 (19.8) | 55 (23.7) | -3.9 | -10.1 | 1.9 | 0.186 |
| **Solid organ transplantation** | 19 (5.1) | 8 (10.5) | -5.4 | -13.6 | 1.3 | 0.074 |
| **Hematopoietic stem cell transplantation** | 7 (1.9) | 0 (0.0) | 1.9 | -2.0 | 3.8 | 0.226 |
| **HIV/Aids** | 16 (1.7) | 2 (0.9) | 0.8 | -1.2 | 2.2 | 0.372 |
| **Malignancy** | 174 (28.9) | 50 (21.6) | 7.3 | 1.0 | 13.1 | 0.026 |
| **Metastatic malignancy** | 100 (10.6) | 18 (7.8) | 2.8 | -1.5 | 6.5 | 0.209 |
| **Hematological condition** | 111 (11.7) | 41 (17.7) | -6.1 | -11.6 | -0.9 | 0.013 |
| **Liver disease** | 153 (16.2) | 13 (5.6) | 10.6 | 6.4 | 14.1 | <0.001 |
| **Chronic kidney disease** | 206 (21.7) | 63 (27.3) | -5.6 | -12.0 | 0.6 | 0.071 |
| **Patient on dialysis** | 24 (2.5) | 11 (4.8) | -2.2 | -5.5 | 0.5 | 0.073 |
| **Chronic pulmonary disease** | 162 (17.2) | 54 (23.3) | -6.1 | -12.2 | -0.3 | 0.032 |
| **Dyslipidemia** | 54 (14.7) | 12 (15.8) | -1.1 | -10.8 | 7.2 | 0.803 |
| **Diabetes mellitus** | 202 (21.2) | 48 (20.7) | 0.5 | -5.5 | 6.2 | 0.854 |
| **Hypertension** | 305 (32.2) | 78 (33.8) | -1.6 | -8.5 | 5.1 | 0.643 |
| **Vascular disease** | 160 (16.9) | 45 (19.5) | -2.6 | -8.4 | 2.8 | 0.349 |
| **Ischaemic stroke** | 50 (5.3) | 11 (4.8) | 0.5 | -3.0 | 3.4 | 0.753 |
| **Dementia** | 12 (1.3) | 1 (0.4) | 0.8 | -0.9 | 1.9 | 0.277 |
| **Coronary disease** | 180 (19.1) | 45 (19.4) | -0.3 | -6.2 | 5.2 | 0.909 |
| **Atrial fibrillation** | 56 (5.9) | 13 (5.6) | 0.3 | -3.4 | 3.4 | 0.849 |
| **Peptic ulcer** | 48 (5.1) | 7 (3.1) | 2.0 | -1.1 | 4.5 | 0.194 |
| **CCI** | 4 (2 - 6) | 4 (2 - 5) |  |  |  | 0.017 |

Values are presented as n (%) or median (IQR). CCI: Charlson Comorbidity Index.

**Table S4: Organ support variables: table.**

|  |  |  |  | **95%CI** | |  |
| --- | --- | --- | --- | --- | --- | --- |
|  | **S3+** | **S2+/S3-** | **Absolute difference** | **lower bound** | **upper bound** | **p** |
| **Mechanical ventilation** | 689 (71.4) | 124 (53.2) | 18.2 | 11.2 | 25.2 | <0.001 |
| **Hemodialysis** | 227 (23.5) | 27 (11.6) | 11.9 | 6.7 | 16.6 | <0.001 |
| **Triple organ support** | 198 (20.5) | 23 (9.9) | 10.6 | 5.7 | 15.0 | <0.001 |
| **Duration of norepinephrine administration** | 2.3 (1.1 - 4.0) | 1.4 (0.7 - 2.7) |  |  |  | <0.001 |
| **Hydrocortisone** | 527 (54.6) | 57 (24.5) | 30.1 | 23.6 | 36.3 | <0.001 |
| **Vasopressine** | 105 (10.9) | 2 (0.9) | 10.0 | 7.2 | 12.1 | <0.001 |
| **Dobutamine** | 164 (17.0) | 18 (7.7) | 9.3 | 4.8 | 13.2 | <0.001 |
| **Epinephrine** | 153 (15.9) | 7 (3.0) | 12.9 | 9.3 | 15.8 | <0.001 |
| **Milrinone** | 125 (13.0) | 12 (5.2) | 7.8 | 3.9 | 11.1 | 0.001 |

Values are presented as n (%) or median (IQR). MOF: multiple organ failure (combination of vasopressor therapy, renal replacement therapy and mechanical ventilation).

**Table S5: Maximal and minimal values of several organ dysfunction varables.**

|  | **S3+** | **S2+/S3-** | **p** |
| --- | --- | --- | --- |
| **SOFA (max)** | 13 (10 - 16) | 10 (8 - 12.5) | <0.001 |
| **Bilirubine (max) (mg/dl)** | 1.4 (0.8 - 3.5) | 0.8 (0.5 - 1.4) | <0.001 |
| **CRP (max) (mg/dl)** | 252.1 (140.9 - 345.3) | 233.3 (148.5 - 323.8) | 0.282 |
| **INR (max)** | 1.7 (1.4 - 2.3) | 1.3 (1.2 - 1.5) | <0.001 |
| **Lactate (max) (mmol/l)** | 4.8 (3.0 - 8.9) | 1.6 (1.3 - 1.8) | <0.001 |
| **Thrombocytes (min) (1000/µl)** | 100 (35 - 168) | 165 (92 - 239) | <0.001 |
| **Leukocytes (max) (1000/µl)** | 18.7 (11.2 - 27.5) | 15.7 (10.8 - 21.4) | <0.001 |
| **Leukocytes (min) (1000/µl)** | 8.6 (3.7 - 14.0) | 9.4 (5.6 - 12.9) | 0.220 |

Values are presented as median (IQR). SOFA: sequental organ failure assessment. CRP: C-reactive protein. INR: International Normalized Ratio.

**Figure S2: Comparison of several organ dysfunction variable between S2+/S3+ and S2+/S3-.**


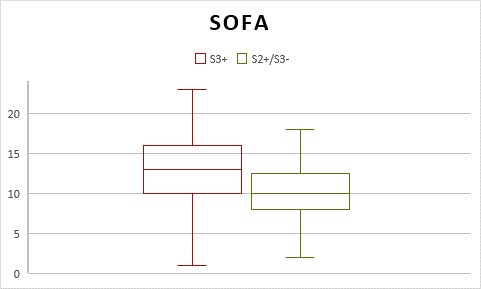


P<0.001


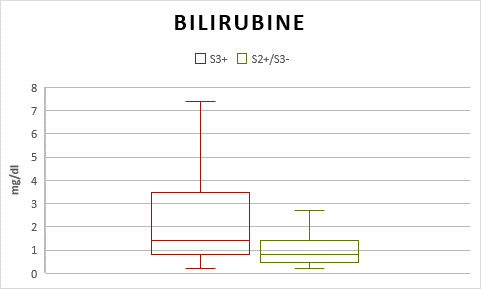


P<0.001


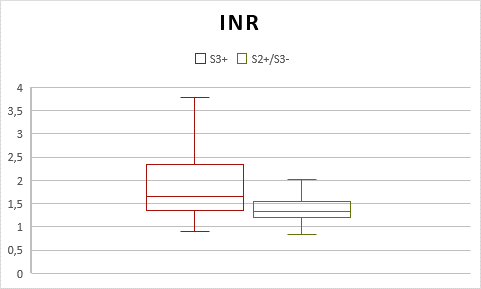


P<0.001


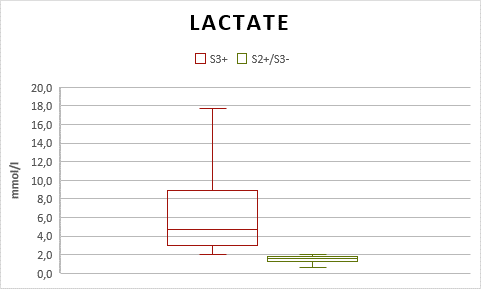


P<0.001


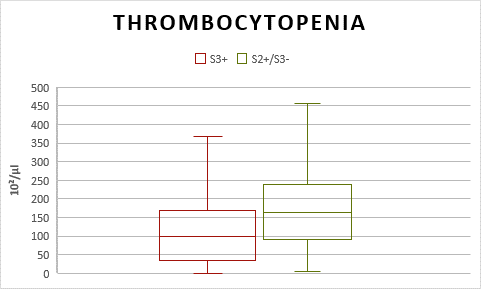


P<0.001


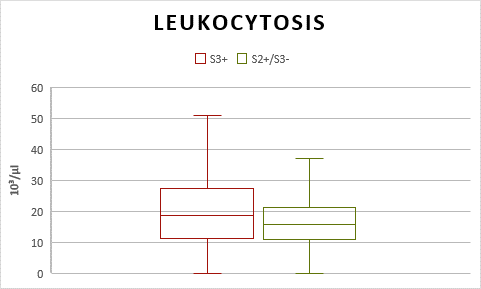


P<0.001

**Figure S3: Evolution of mortality of S3+ patients.**

Annual evolution of number of patients with septic shock according to the Sepsis-3 definition, expressed as proportion of total patients admitted to the ICU (bars). ICU and hospital mortality of patients with septic shock according to the Sepsis-3 definitions remain relatively constant over the years (lines).

**Table S6: Comparison between septic shock patients with or without medical admission reason.**

|  | **Medical admission reason** | **Non medical admission reason** |  | **95%CI** | | **p** |
| --- | --- | --- | --- | --- | --- | --- |
|  | (n = 621) | (n = 577) | Absolute difference | lower bound | upper bound |  |
| **ICU mortality** | 256 (42.2) | 203 (36.1) | 6.1 | 0.5 | 11.6 | 0.034 |
| **Hospital Mortality** | 325 (55.1) | 241 (46.2) | 8.9 | 3.1 | 14.8 | 0.003 |
| **Lactate** | 3.4 (2.0 - 6.9) | 4.2 (2.5 - 8.0) |  |  |  | <0.001 |
| **SOFA** | 12 (9 - 16) | 12 (10 - 16) |  |  |  | 0.741 |

Values are expressed as n (%) or median (IQR).

Comparison between patients with or without a medical admission reason in the baseline Sepsis-2 septic shock population (S3+ and S2+/S3-). Lactate levels are lower in patients with a medical admission reason, which explains that these patients are more often excluded from the Sepsis-3 definition of septic shock. Both ICU and hospital mortality are larger in patients with a medical admission reason compared to patients admitted for non medical reasons although there is no difference in in severity of the disease according to the SOFA score.

**Table S7: Comparison between septic shock patients with or without respiratory infection.**

|  | **Respiratory infection** | **Non respiratory infection** |  | **95%CI** |  | **p** |
| --- | --- | --- | --- | --- | --- | --- |
|  | **(n = 383)** | **(n = 815)** | **Absolute difference** | **lower bound** | **upper bound** |  |
| **ICU mortality** | 175 (47.3) | 284 (35.5%) | 11.8 | 5.7 | 17.8 | <0.001 |
| **Hospital Mortality** | 200 (56.2) | 366 (48.4) | 7.6 | 1.4 | 13.9 | 0.016 |
| **Lactate** | 3.0 (2.0 - 6.2) | 4.2 ( 2.4 - 8.0) |  |  |  | <0.001 |
| **SOFA** | 12 (10 - 16) | 12 (9 - 16) |  |  |  | 0.433 |

Values are expressed as n(%) or median (IQR).

Comparison between patients with a respiratory infection as cause for septic shock and those with another infection causing it in the baseline Sepsis-2 septic shock population (S3+ and S2+/S3-). Patients with a respiratory infection had lower lactate levels, which is an explanation why they less often fullfil the Sepsis-3 septic shock criteria. Patients with a respiratory infection as cause for septic shock have higher ICU and hospital mortality despite a similar SOFA score as patients with other infections as cause for septic shock.

**Table S8: Comparison between septic shock patients with or without chronic liver disease.**

|  | **Liver disease** | **No liver disease** |  | **95%CI** |  | **p** |
| --- | --- | --- | --- | --- | --- | --- |
|  | **(n = 166)** | **(n = 1012)** | **Absolute difference** | **lower bound** | **upper bound** |  |
| **ICU mortality** | 92 (56.4) | 358 (36.3) | 20.1 | 11.9 | 28.2 | <0.001 |
| **Hospital Mortality** | 115 (71.4) | 440 (47.2) | 24.2 | 16.2 | 31.6 | <0.001 |
| **Lactate** | 5.4 (3.0 - 10.8) | 3.5 (2.1 - 6.8) |  |  |  | <0.001 |
| **SOFA** | 15 (12 - 18) | 12 (9 - 15) |  |  |  | <0.001 |

Values are expressed as n(%) or median (IQR).

Comparison between patients with septic shock and chronic liver disease and patients with septic shock without liver disease in the baseling Sepsis-2 septic shock population.

Patients with chronic liver disease have higher lactate levels compared to those without chronic liver disease making it more likely to fullfil the Sepsis-3 shock criteria. ICU and hospital mortality are higher in the group of patients with chronic liver disease, as is the SOFA score.

**Table S9: Comparison in patients fullfilling Sepsis-3 citeria (S3+) and patients not fullfilling Sepsis-3 criteria (S2+/S3-) in the subgroup of patients with chronic liver disease.**

|  |  |  |  | **95%CI** | |  |
| --- | --- | --- | --- | --- | --- | --- |
|  | **Liver disease S3+**  **(n=153)** | **Liver disease S2+/S3-**  **(n=13)** | **Absolute difference** | **lower bound** | **upper bound** | **p** |
| **ICU mortality** | 91 (60.7) | 1 (7.7) | 53.0 | 28.3 | 66.1 | <0.001 |
| **Hospital mortality** | 111 (75.0) | 4 (30.8) | 44.2 | 16.5 | 66.2 | 0.001 |
| **Organ support** |  | | | | | |
| **Mechanical ventilation** | 106 (69.3) | 9 (69.2) | 0.1 | -22.6 | 27.3 | 0.997 |
| **Renal replacement therapy** | 49 (32.0) | 2 (15.4) | 16.6 | -9.3 | 33.8 | 0.212 |
| **Triple organ support** | 41 (26.8) | 2 (15.4) | 11.4 | -14.3 | 28.5 | 0.367 |
| **Hydrocortisone** | 83 (54.2) | 3 (23.1) | 31.2 | 3.8 | 51.2 | 0.031 |
| **Dobutamine** | 13 (8.5) | 0 (0) | 8.5 | -11.0 | 15.8 | 0.274 |
| **Vasopressine** | 18 (11.8) | 0 (0) | 11.8 | -8.0 | 19.2 | 0.19 |
| **Adrenaline** | 15 (9.8) | 1 (7.7) | 2.1 | -20.9 | 14.8 | 0.804 |
| **Milrinone** | 10 (6.5) | 0 (0) | 6.5 | -12.8 | 13.7 | 0.342 |
| **Disease severity markers** |  | | | | | |
| **Bilirubine** | 5.9 (2.7 - 12.1) | 1.3 (1.0 - 3.9) |  |  |  | 0.002 |
| **INR** | 2.3 (1.7 - 3.8) | 1.3 (1.2 - 1.5) |  |  |  | <0.001 |
| **Thrombocyte count** | 52 (25 - 106.3) | 56 (37.5 - 147) |  |  |  | 0.385 |
| **CRP** | 104.2 (58.0 - 203.3) | 165.4 (122 - 255.4) |  |  |  | 0.038 |
| **Leukocyte count** | 18.7 (11.1 - 28.5) | 9.4 (6.9 - 27.5) |  |  |  | 0.147 |
| **Lactate level** | 6.3 (3.7 - 11.1) | 1.5 (1.2 - 1.8) |  |  |  | <0.001 |
| **SOFA** | 16 (12 - 18) | 12.5 (9 - 13.75) |  |  |  | 0.003 |

Values are expressed as n(%) or median (IQR). Triple organ support: combination of vasopressor therapy, mechanical ventilation and renal replacement therapy. INR: International Normalized Ratio. CRP: C-reactive protein. SOFA: sequential organ failure assessment.

Patients with liver disease who fullfil the Sepsis-3 shock criteria (S3+) have higher ICU and hospital mortality compared to those that only fullfil the Sepsis-2 shock criteria (S2+/S3-). Except for the admission of hydrocortisone, there is no difference in organ support between both groups.

Several markers of disease severity are different between the S3+ and S2+/S3- group: there is a higher bilirubine level, higher INR and lower CRP levels in the S3+ group. This is not surprising since derangement of these values can be caused by deterioration of a previously compromised liver function. Lactate levels are higher in S3+ patients since this is part of the definition. SOFA score is also higher in S3+ liver disease patients. One fourth of patients in the S2+/S3- group has a bilirubin value of 1.3 mg/dl or less (0 or 1 points on the liver SOFA subscore) while one fourth of patients in the S2+/S3+ group has bilirubine value of more than 5.9 mg/dl (3 points on the liver SOFA subscore).

**Figure S4: Proportion of S3+ patients and mortality in function of lactate levels.**

Blue: change in proportion of patients satisfying the Sepsis-2 septic shock definition that would satisfy the Sepsis-3 septic shock definition (= S3+ patients) if the lactate criterion would be increased in 1 mmol/l steps comply with the definition. Orange: evolution of Sepsis-3 septic shock hospital mortality if the lactate criterion would be increased in 1 mmol/l steps. For example: if the lactate criterion would be 5 mmol/l to satisfy the definition of septic shock, then 38.1% of the “old” S2+ patients would be identified as having septic shock and these patients would have a hospital mortality of 68.3%.

**Figure S5: Proportion of patients with chronic liver disease in the S2+/S3- and S3+ group in function of lactate levels.**

Change in proportion of patients with chronic liver disease (CLD) in the S3+ and S2+/S3- group if the lactate criterion would be increased in 1 mmol/l steps to comply with the definition. For example: if the lactate criterion would be 5 mmol/l to satisfy the definition of septic shock, the proportion of patients with chronic liver disease would be 19.8% in the S3+ group and 10.6% in the S2+/S3- group. This difference remains significant with each incremental step in lactate levels (all p < 0.001). So there is no lactate level at whih the imbalance in proportion of patients with chronic liver disease between the two groups would disappear.
